# Temperatures and precipitation affect vegetation dynamics on Scandinavian extensive green roofs

**DOI:** 10.1007/s00484-020-02060-2

**Published:** 2020-12-11

**Authors:** Joel Lönnqvist, Hans Martin Hanslin, Birgitte Gisvold Johannessen, Tone Merete Muthanna, Maria Viklander, Godecke Blecken

**Affiliations:** 1 Department of Civil, Environmental and Natural Resources Engineering, Luleå University of Technology, Luleå, Sweden; 2 Department of Urban Greening and Vegetation Ecology, Norwegian Institute of Bioeconomy Research, Ås, Norway; 3 Department of Civil and Environmental Engineering, Norwegian University of Science and Technology, Trondheim, Norway

**Keywords:** Green roof, Mean temperature, Precipitation, Freeze-thaw cycles, Survival, Spontaneous vegetation

## Abstract

Standard succulent vegetation mixes developed mostly in temperate climates are being increasingly used on green roofs in different climate zones with uncertain outcome regarding vegetation survival and cover. We investigated vegetation on green roofs at nine temperate, cold, and/or wet locations in Norway and Sweden covering wide ranges of latitude, mean annual temperature, annual precipitation, frequencies of freeze-thaw cycles, and longest annual dry period. The vegetation on the roofs were surveyed in two consecutive years, and weather data were compiled from meteorological databases. At all sites we detected a significant decline in species compared to originally intended (planted/sown) species. Both the survival rate and cover of the intended vegetation were positively related to the mean annual temperature. Contrary to a hypothesis, we found that intended vegetation cover was negatively rather than positively related to mean annual precipitation. Conversely, the unintended (spontaneous) vegetation was favoured by high mean annual precipitation and low mean annual temperature, possibly by enabling it to colonize bare patches and outcompete the intended vegetation. When there is high mortality and variation in cover of the intended vegetation, predicting the strength of ecosystem services the vegetation provides on green roofs is difficult. The results highlight the needs for further investigation on species traits and the local factors driving extinction and colonizations in order to improve survivability and ensure a dense vegetation throughout the successional stages of a green roof.

**Supplementary Information:**

The online version contains supplementary material available at 10.1007/s00484-020-02060-2.

## Introduction

Green roofs are becoming increasingly popular in urban areas, partly because of their architectural values and partly due to their potential multi-functionality (Dusza et al. 2017). They can make particularly important contributions to restoration of some of the ecosystem services lost through construction of buildings in densely populated areas (Getter and Rowe 2006; Oberndorfer et al. 2007; Lundholm 2015). One of the most widely recognized ecosystem services they provide is the ability to retain and attenuate stormwater runoff, thereby potentially decreasing strain on stormwater sewers, risks of combined sewer overflows, eroded material in receiving waters, and flooding (Stovin et al. 2015). Although several parts of a green roof contribute to the functions, such as the substrate and water-holding layers, the plant cover plays significant roles in the following: stormwater retention (VanWoert et al. 2005; Stovin et al. 2015); urban air cooling (MacIvor et al. 2018; Speak et al. 2013); delivering urban biodiversity, relative to conventional roofs (Williams et al. 2014); and buildings’ thermal regulation (Sailor 2008).

However, in efforts to ensure the establishment of dense, persistent vegetation cover, to a large extent the industry relies on *Sedum* species that grow naturally in shallow substrates, tolerate long periods of drought, and form dense ground cover (Dvorak and Volder 2010). Their lack of requirement for deeper substrates also helps to keep building loads down (Durhman et al. 2007), an especially attractive feature in areas with cold climates and potentially heavy snow loads in winter.

The optimal green roof vegetation should provide dense cover and have high water use when water is abundant and low water use when it is scarce, but since this is a rare combination of features, compromises are usually required (Farrell et al. 2013). Species with greater height and biomass, more extensive root systems, and higher transpiration rates may make stronger contributions to stormwater management than *Sedum* spp. (Lundholm et al. 2010; Farrell et al. 2013). However, in cold and wet climates, the potential of non-succulent vegetation on green roofs has been questioned due to the increased risk of permanent wilting unless substrate storage volumes are increased considerably. On the other hand, *Sedum* has been found to outperform meadow vegetation in terms of air cooling (MacIvor et al. 2016) and can potentially facilitate the establishment and survival of other plant taxa (Butler and Orians 2011). Planting mixtures of species with complementary traits is frequently advised in green roof literature as a means to improve overall function (Lundholm et al. 2010; Cook-Patton and Bauerle 2012). A major complication is that the success of the designed and/or (originally) planted vegetation (hereafter referred to as *intended* vegetation) can vary substantially, since green roofs are subject to successional changes involving species extinctions, spontaneous colonizations (hereafter referred to as *unintended* vegetation), and changes in species’ abundance over time (Dunnett et al. 2008; Lönnqvist et al. submitted). The performance of the plant communities used, and both the nature and magnitude of successional changes, will depend on the local climatic conditions, and the successional changes in the vegetation will affect green roofs’ functions through changes in factors such as albedo and evapotranspiration rates (Speak et al. 2013). Thus, it is important to ensure that green roof vegetation is adapted for the particular local conditions (Monterusso et al. 2005; Getter et al. 2009).

Some of the earliest known examples of green roofs were ‘sod roofs’ (also called turf roofs) which were popular in medieval Scandinavia, as a response to harsh climate and resource scarcity; later the technique spread with migrants to Iceland and North America. The vegetation of traditional Scandinavian sod roofs reflected the local flora at sites of the originally harvested turf (Jim 2017). More recently, modern extensive green roofs have started to be installed in Scandinavian urban areas. These roofs were first developed in central Europe as a systematically vegetated form of the spontaneously colonized tar-paper roofs of Germany (Köhler and Poll 2010). The vegetation used in these roofs was originally sourced from predominantly temperate climate zones in central Europe and rock outcrop–type habitats in various parts of the world. While traditional sod roofs are still popular in Norway, extensive roofs with pre-established sedum mats are the most common type of modern green roofs used in urban areas in Norway (Braskerud 2014) and in Sweden (Emilsson and Rolf 2005).

Due to the inevitable effects of local climate on green roofs’ performance and succession, as green roof technology spreads to new geographical areas, there are clear needs for greater understanding of the relations between their species composition and performance in specific climatic conditions. Green roof vegetation has been studied extensively in the temperate climate zones where they originated, but received little attention elsewhere (Vasl et al. 2017). Previous studies in Fennoscandia have studied the importance of age and substrate depth in shaping plant communities (Gabrych et al. 2016) and the means of establishment (Emilsson and Rolf 2005), and studies performed in southern Sweden’s humid continental climate (Dfb) found succulent vegetation cover to decrease over a 3-year period and the moss cover increased significantly (Emilsson 2008). Since the same species mixtures are often used across broad geographical areas, there is a need to understand how local weather and climate affect their dynamics (Tran et al. 2019). Our study includes sites located in humid continental (Köppen Dfb), temperate oceanic (Köppen Cfb), and high-latitude subarctic climates (Köppen Dfc) which provides an opportunity to contribute to such understanding. We investigated the performance of the vegetation of young (2–8 years old) green roof systems at nine locations with widely varying climates in Norway and Sweden. We surveyed the vegetation on these roofs in two consecutive years and evaluated responses of both the intended and unintended vegetation, in terms of survival and cover, to green roof design parameters and local weather variables. Before the study, we formulated the following hypotheses, based on relevant literature:

1. The standard *Sedum* vegetation would not perform optimally in northern climatic conditions, and that survival rates and cover of standard green roof vegetation would be positively related to mean annual temperatures and annual precipitation.
2. That both frequencies of freeze-thaw cycles and lengths of dry periods would significantly affect vegetation performance.

## Methods

### Locations, roof characteristics, and experimental design

**Fig. 1:**
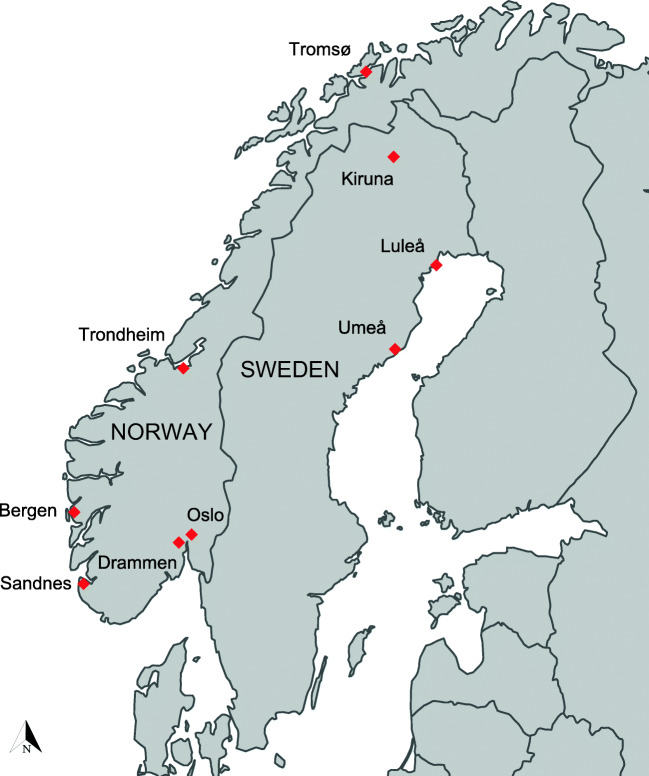
Locations of the study sites in Scandinavia in northern Europe

Forty-two different roofs were surveyed across nine locations (Table 1). Locations in Norway (*N* = 6) were deliberately selected to cover much of the national climatic gradients relevant for urban green roofs. At each of these locations, a set of different green roof solutions was applied in experimental plots on actual roofs (with treatments side by side in a randomized design), to compare the performance of their vegetation across the climatic range. Surveyed roofs in Sweden (*N* = 10) were full-scale green roofs covering tops of buildings in residential areas and industrial zones located in the colder northern part of the country. Generally, mean temperatures decrease with increasing latitude of the sites, and precipitation is lower at the Swedish sites than at the Norwegian sites (Fig. 1 and Table 1).

**Table 1:** Key properties of the surveyed green roofs. Roofs located in Sandnes, (S), Drammen (D), Oslo (O1 and O2), Bergen (B), Trondheim (Tm), and Tromsø are in Norway, while those in Umeå (U1), Luleå, (L1 and L2), and Kiruna (K1 and K2) are in Sweden. WHL and Substrate d refer to depths of the water-holding layer and substrate, respectively. Roofs are ordered top-bottom by rising latitude

Roof	Latitude	Longitude	WHL (mm)	Substr. d (mm)	Aspect	Slope (°)	Age (years)	Area (m^2^)	Originally intended species (from suppliers lists)	Species mix
S-1	58.87	5.76	10	30	N-NE	15	2	85	6	1
S-2	58.87	5.76	5	55	N-NE	15	2	85	5	3
S-3	58.87	5.76	10	30	N-NE	15	2	85	4	4
S-4	58.87	5.76	25	30	N-NE	15	2	85	7	2
D-1	59.74	10.2	10	30	SE-NW	20	2	42	6	1
D-2	59.74	10.2	10	30	SE-NW	20	2	42	6	2
D-3	59.74	10.2	5	55	SE-NW	20	2	42	5	3
D-4	59.74	10.2	10	30	SE-NW	20	2	42	6	1
D-5	59.74	10.2	25	30	SE-NW	20	2	42	7	2
D-6	59.74	10.2	5	55	SE-NW	20	2	42	5	3
D-8	59.74	10.2	10	30	SE-NW	20	2	42	4	5
D-9	59.74	10.2	10	30	SE-NW	20	2	42	6	1
D-11	59.74	10.2	25	30	SE-NW	20	2	42	7	5
D-12	59.74	10.2	10	30	SE-NW	20	2	42	3	1
O1-1	59.91	10.8	0	30	SW	0	2	77	6	1
O1-2	59.91	10.8	4	40	SW	0	2	77	5	3
O1-3	59.91	10.8	0	40	SW	0	2	77	7	2
O1-4	59.91	10.8	5	30	SW	0	2	77	3	5
O2-1	59.96	10.73	10	30	N	3	7	80	6	1
O2-2	59.96	10.73	4	40	N	3	8	80	5	3
O2-3	59.96	10.73	10	30	N	3	7	80	6	1
B-1	60.38	5.33	10	30	E	15	2	77	6	1
B-2	60.38	5.33	25	30	E	15	2	77	7	2
B-3	60.38	5.33	5	85	E	15	2	77	5	3
B-4	60.38	5.33	10	30	E	15	2	77	4	4
B-5	60.38	5.33	3.1	60	E	15	2	77	4	4
Tm-1	63.41	10.41	10	30	E	9	2	148	6	1
Tm-2	63.41	10.41	25	30	E	9	2	148	7	2
Tm-3	63.41	10.41	5	55	E	9	2	148	5	3
U1-15	63.81	20.29	10	48	S	12	2	1600	12	8
U1-19	63.81	20.29	10	46	S	12	2	1640	12	8
U1-2	63.81	20.29	10	52	N	15	2	875	12	8
U1-5	63.81	20.29	10	58	S	12	2	1485	12	8
L1-1-N	65.58	22.16	10	31	N	10	4	5520	8	6
L1-1-S	65.58	22.16	10	25	S	10	4	2511	8	6
L1-2-N	65.58	22.16	10	25	N	5	4	1068	8	6
L2-1-S	65.58	22.16	10	23	S	12	3	84	8	7
K1	67.86	20.22	10	33	N	6	2	384	8	6
K2	67.86	20.22	10	32	S	6	2	384	8	6
To-2	69.65	18.94	4	40	SW	0	2	90	5	3
To-3	69.65	18.94	0	40	SW	0	2	90	7	2
To-4	69.65	18.94	10	30	SW	0	2	90	4	4

As shown in Table 1, all surveyed roofs were thin-substrate *Sedum*-based extensive green roofs. Most were 2–3 years old at the time of the first survey in 2016, but a few older roofs (4–8 years old) were also included. The roofs were planted with different standard mixtures delivered from Swedish and/or Norwegian suppliers. Originally eight vegetation mixtures were planted on the roofs, but several of these only differ in the inclusion or exclusion of one or a few species (Table 2). The roofs were established by pre-vegetated mats or pre-vegetated mats complemented with cuttings. The roofs received standard maintenance such as fertilization, as recommended by the supplier.

**Table 2:** The species mixes used for the intended vegetation (for roofs receiving each of the mixes, see Table 1). The species included in each mix—largely according to the Swedish taxonomic database SKUD (Swedish University of Agriculture)—are indicated by asterisks. *Phedimus coll* includes *Phedimus hybridus* and *Phedimus kamtschaticus* due to difficulties in differentiating them

Species	Mix 1	Mix 2	Mix 3	Mix 4	Mix 5	Mix 6	Mix 7	Mix 8
*Festuca ovina*								*
*Sedum acre*	*	*	*	*	*	*	*	*
*Sedum album*	*	*	*	*	*	*	*	*
*Sedum anglicum*					*			
*Hylotelephium cyaneum*								*
*Sedum hispanicum*		*						*
*Sedum lydium*		*	*					*
*Sedum oreganum*								*
*Sedum rupestre*		*	*	*			*	*
*Sedum sexangulare*	*	*	*			*	*	*
*Sedum pulchellum*	*					*		
*Sedum spurium*			*	*		*	*	*
*Hylotelephium ewersii*	*					*	*	*
*Phedimus coll*	*	*	*	*		*	*	*
Total number of species	6	7	7	6	3	7	7	12

### Survey methods

We monitored vegetation cover and species presence/absence in permanent 1 × 1 m quadrats placed in transects with evenly spaced quadrats reflecting the surface area of the roof and avoiding edge zones and shaded areas. The total intended vegetation cover, total unintended vegetation cover, total moss cover, and total bare vegetation cover as well as the percentage cover of each individual intended vascular plant species were recorded in each quadrat. Each quadrat was divided into smaller 0.1 × 0.1 m squares, corresponding to 1% cover, to facilitate estimation of plant cover. The exact locations of quadrats were recorded so that successional changes at the same spots could be monitored by surveys in consecutive years. Due to the difficulties of differentiating *Phedimus hybridus* from *P*. *kamtschaticus* when not in flower, the two species were merged and are referred to as *P*. *coll* hereafter.

### Weather variables

Weather time series for the years prior to the surveys were collected from the Norwegian Meteorological Institute (MET Norway) and Swedish Meteorological and Hydrological Institute (SMHI) as daily averages for precipitation and 6-h averages for temperature. To acquire complete full time series of data for all sites, weather data from several weather stations in the same town were merged when data were incomplete. All weather variables were compiled from July 15 until the same date of the following year when surveys were conducted. Since relevant weather indices are often highly correlated (Johannessen et al. 2017), we selected a subset to represent major gradients. Freeze-thaw cycles were defined as all changes between negative and positive temperatures recorded with 6-h resolution, irrespective of snow cover since such data were incomplete. The duration of the longest drought episode was defined as the longest consecutive sequence of days without precipitation (recorded in days), and mean annual temperature as the mean temperature recorded between July 15 and July 15 the following year. Total precipitation was the total precipitation during the time from July 15 in the preceding year until July 15 in the year of the survey. Samples from 2016 and 2017 were treated as separate replicates to account for the variability in weather and vegetation between the years.

### Statistical analysis

We used generalized linear models (GLMs) with binomial family and logit link function (Warton and Hui 2011) in R (version 3.6.1) to explore the relationships between vegetation, weather, and roof design parameters. The functions were implemented in the MASS package with vegetation covers and success of the intended vegetation set as response variables and weather parameters, water-holding layer and substrate depths, and roof slope as predictors. Paired Wilcoxon pairwise comparisons and analysis of variance (ANOVA) were applied with the MASS package in R to compare numbers of intended species detected in surveys (R code in electronic supplementary material).

## Results

### Weather during the year preceding the vegetation surveys

Mean temperature generally declines with increasing latitude (Table 3). However, Tromsø (the northernmost location of surveyed roofs) has a milder climate than Kiruna and Luleå, due to oceanic influence. Across locations, 2017 was a significantly colder year than 2016 (*p* < 0.01). Accumulated precipitation, duration of the longest drought, and frequencies of freeze-thaw cycles also differed between years, but there was no significant difference in vegetation cover between the years (*p* > 0.05, in electronic supplementary material). Frequencies of freeze-thaw cycles varied between years and were lowest at the rainiest locations, Sandnes and Bergen, both of which have an oceanic (Köppen Cfb) climate. Bergen received significantly more precipitation (Table 3) than the other sites of surveyed roofs, and the locations in northern Sweden received the least.

**Table 3:** Summary of weather during the 12 months before the 15th of July in indicated years at sites of the surveyed roofs. Freeze-thaw cycles refer to shifts between negative and positive temperature with 6-h resolution. The longest dry period is the longest series of consecutive days with no registered precipitation

Location	Latitude	Köppen climate zone	Year	Freeze-thaw cycles	Mean temperature (°C)	Total precipitation (mm)	Longest dry period (days)
Sandnes	58.87	Cfb	2017	26	8.7	1195	13
Sandnes	58.87	Cfb	2016	31	8.9	1183	13
Drammen	59.74	Dfb	2017	65	7.3	681	10
Drammen	59.74	Dfb	2016	69	7.2	845	19
Oslo	59.91	Dfb	2017	65	7.4	709	8
Oslo	59.91	Dfb	2016	45	7.5	882	16
Bergen	60.38	Cfb	2017	28	8.8	2740	15
Bergen	60.38	Cfb	2016	29	8.9	2386	8
Trondheim	63.41	Cfb	2017	63	6.1	1084	23
Trondheim	63.41	Cfb	2016	53	6.5	832	21
Umeå	63.81	Dfb	2016	53	5.3	520	21
Luleå	65.58	Dfc	2016	60	3.7	666	20
Kiruna	67.86	Dfc	2016	49	0.9	523	14
Tromsø	69.65	Dfc	2017	71	3.7	1151	9
Tromsø	69.65	Dfc	2016	62	4.4	1015	9

### Vegetation cover across locations

**Fig. 2:**
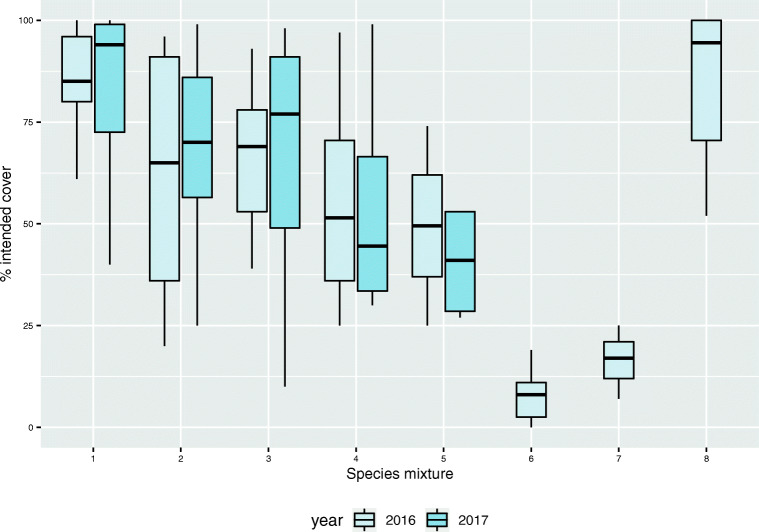
Box-plot of intended vegetation cover obtained with each species mix (as listed in Table 2) across years and locations. Roofs with species mixes 6 and 7 were only surveyed in 2016. The median, 25th, and 75th percentile hinges and 1.5 inter-quartile range whiskers are shown

**Fig. 3:**
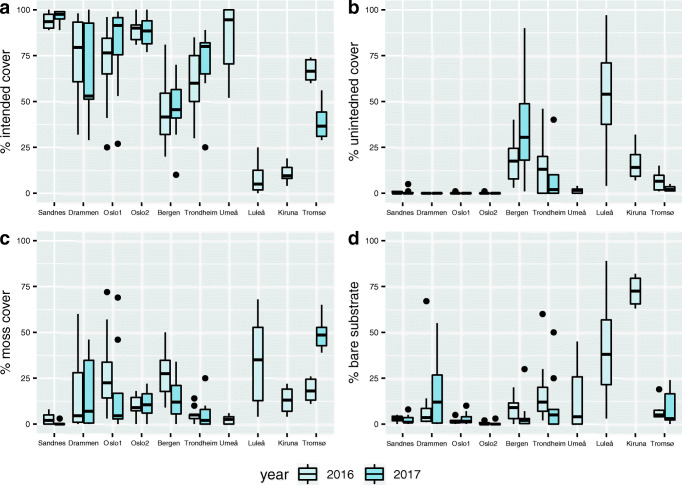
Cover of vegetation and bare substrate on roofs at each of the 10 sites (southernmost to northernmost from left to right). The median, 25th, and 75th percentile hinges, 1.5 inter-quartile range whiskers, and individually plotted outliers are shown

Intended vegetation cover varied depending on species mix (Fig. 2) and location (Fig. 3a). Both Sandnes and Oslo2 had consistently high (> 75%) intended vegetation cover while the other sites showed a more varying cover of the intended vegetation (Fig. 3a). There were also large differences in vegetation cover at sites in the same locations (Fig. 3). Notably, roofs at two of the northerly Swedish sites (Luleå and Kiruna) had the lowest vascular plant cover and highest cover of bare substrate (Fig. 3d). Moss cover varied, but the most northerly roofs (at Tromsø) had the greatest moss cover, and correspondingly low intended vegetation cover. Bare substrate cover was highest on roofs at Kiruna, where the annual mean temperatures were lowest (Fig. 3), and unintended species contributed more to the total vascular plant cover on roofs in Bergen and Trondheim than in other sites.

### Changes in species composition over time

**Fig. 4:**
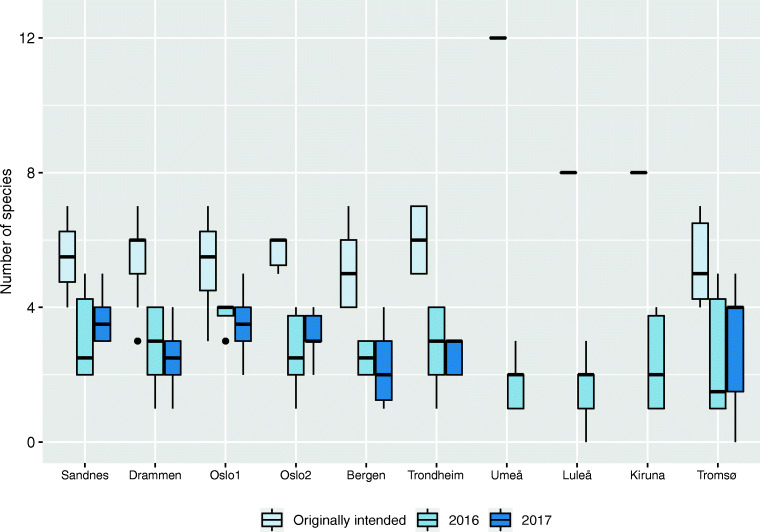
Numbers of species originally intended for the roofs and numbers of species found during surveys. Within each of the Swedish sites (Umeå, Luleå, and Kiruna), the roofs had the same intended species composition and these roofs were only surveyed in 2016. The species originally intended for the roofs were all *Sedum* and other succulents, except in Umeå where the grass *F*. *ovina* was also planted. The median, 25th, and 75th percentile hinges, 1.5 inter-quartile range whiskers, and individually plotted outliers are shown

Despite the roofs’ relatively young age, we found a clear filtering of species composition over time, as we did not detect about half of the original species, which thus either failed to establish or rapidly declined at all sites (Table 3). At all sites there were significantly lower numbers of species, according to paired Wilcoxon pairwise comparisons (*p* < 0.05, Fig. 4), than the originally intended composition, except in Tromsø, where there was no significant difference between the originally intended number of species and number of species detected in 2016 (paired Wilcoxon’s pairwise comparison: *p* = 0.063; Fig. 4). There was no significant difference in numbers of species found in 2016 and 2017 (paired Wilcoxon pairwise comparisons, *p* > 0.05). *Phedimus coll* (composed of the two species *P*. *kamtschaticus* and *P*. *hybridus*) was found on roofs at all sites except Kiruna in Sweden and had the highest overall mean cover (19%), followed by *S*. *acre* (15%), *S*. *album* (11%), and *S*. *spurium* (3.2%). *Sedum anglicum*, *S. forsterianum*, *H*. *cyaneum*, *S*. *oreganum*, *S. reflexum*, and *H*. *telephium* were included in some original species compositions but were not detected in the surveys (Tables 4 and 5).

**Table 4:** Intended species, and numbers of plots where they were planted and refound (i.e. detected in our surveys)

**Species**	***S*.** ***anglicum***	***S*.** ***album***	***P*.** ***coll***	***F*.** ***ovina***	***S*.** ***acre***	***S*.** ***ewersii***	***S*.** ***sexangulare***	***S*.** ***spurium***
Plots listed	8	124	117	4	78	44	100	56
Plots refound	8	96	88	3	49	27	50	25
Percent refound	100.0	77.4	75.2	75.0	62.8	61.4	50.0	44.6
**Species**	***S. lydium***	***S. hispanicum***	***S. pulchellum***	***S. cyaneum***	***S. floriferum***	***S. oreganum***	***S. rupestre***	**Total mean**
Plots listed	30	30	39	4	6	4	77	–
Plots refound	8	7	1	0	0	0	0	–
Percent refound	26.7	23.3	2.6	0.0	0.0	0.0	0.0	39.9

**Table 5:** Mean cover (with standard error in brackets) of the intended species on representative green roofs in 2016 and 2017. For meanings of the site abbreviations, see Table 1). *P*. *coll* refers to a combination of *P*. *kamtschaticus* and *P*. *hybridus*

Roof	B-5	D-3	K1	L1	O1–1	O2–2	S-2	Tm-1	To-3	U1–5
Vascular plants	75.7 (± 9.7)	56.5 (± 6.9)	16 (± 1.5)	13 (± 2.5)	85 (± 5.1)	87.5 (± 3.2)	95.7 (± 2.3)	84.7 (± 3.2)	59.8 (± 12.5)	87 (± 9.2)
*F*. *ovina*	0	0	0	0	0 (± 0)	0	0	0	0	1.3 (± 0.8)
*S*. *acre*	10.2 (± 1.8)	0.2 (± 0.2)	2.6 (± 2.1)	3.3 (± 0.8)	0.3 (± 0.3)	6 (± 2)	0.7 (± 0.4)	8.8 (± 3.8)	40.5 (± 6.4)	85.3 (± 8.5)
*S*. *album*	3.2 (± 2.3)	12.8 (± 10.8)	3.3 (± 0.6)	9.3 (± 1.8)	54.3 (± 11.5)	1.7 (± 0.6)	5.5 (± 1.8)	14.1 (± 5.7)	3.2 (± 1.6)	0.3 (± 0.3)
*S*. *anglicum*	0	0	0	0	0	0	0	0	0	0
*S*. *cyaneum*	0	0	0	0	0	0	0	0	0	0
*S*. *forsterianum*	0	0	0	0	0	0	0	0	0	0
*S*. *hispanicum*	0	0	0	0	0	0.5 (± 0.5)	9.5 (± 2)	0	2.7 (± 2.7)	0
*S*. *lydium*	2.7 (± 2.4)	0.7 (± 0.4)	0	0	0	39.5 (± 13.3)	61.2 (± 7)	0.1 (± 0.1)	2 (± 1)	0
*S*. *oreganum*	0	0	0	0	0	0	0	0	0	0
*S*. *reflexum*	0	0	0	0	0	0	0	0	0	0
*S*. *sexangulare*	9.5 (± 2.1)	1.7 (± 1.1)	0	0	1.3 (± 0.3)	16.7 (± 5.2)	1.5 (± 0.9)	0.7 (± 0.7)	0	0
*S*. *pulchellum*	0	0	0	0.3 (± 0.3)	0	0	0	0	0	0
*S*. *spurium*	9.2 (± 4)	5.2 (± 2.2)	0	0	0	0.5 (± 0.5)	0.7 (± 0.7)	6.5 (± 6.2)	0	0
*H*. *telephium*	0	0	0	0	0	0	0	0	0	0
*H*. *ewersii*	0	0	4 (± 2)	0	9 (± 5.1)	0	1.7 (± 1)	7.8 (± 4.1)	0	0
*P*. *coll*	1.2 (± 1.2)	35 (± 8.7)	0	0.3 (± 0.3)	19.6 (± 5.2)	21.8 (± 21.8)	12.7 (± 6.1)	44 (± 9.6)	3.7 (± 2.7)	0

The GLM results showed that abiotic and weather factors influenced vegetation cover and percentage of the originally intended species that were refound, i.e. detected in the surveys (Table 6). Increases in mean annual temperature had significant positive effects on the intended vegetation cover and percentage of originally intended species that were detected in the surveys. Temperature had no significant effect on moss cover, and negative effects on the unintended vegetation and bare substrate cover (Table 6). Increases in total precipitation had significant positive effects on unintended plant cover while it had a significant negative effect on the intended vegetation cover, and no significant effect on the percentage of detected intended species. Increasing frequencies of freeze-thaw cycles had positive effects on both moss and total plant cover, but no other significant effects on vegetation cover. Increases in roof slope had significant positive effects on amount of bare substrate found. No significant effect of variation in depth (water-holding layer and substrate) on either vegetation cover or the proportion of refound species was detected. Increases in duration of the longest drought period had no significant effects on vegetation cover or proportion of refound species. Variables such as roof area and roof age, which had little variation in the dataset, could potentially confound the results of the statistical analysis; however, these variables showed no significant effects when included in the GLMs.

**Table 6:** Results of generalized linear models (GLMs) with binomial family and logit link function, showing responses in the first row and factors in the first column. For responses of vegetation cover, bare substrate, and percentage of refound species, *n* = 144. The variable depth is the thickness of the water-holding layer and substrate, in mm. Freeze-thaw cycles refers to the number of shifts between negative and positive temperature with 6-h resolution. The longest drought is the longest consecutive series of days with no registered precipitation. Significant *p* values (< 0.05) are indicated in italic and positive or negative effects by upward and downward arrows, respectively

	Intended cover	Unintended cover	Moss cover	Bare substrate	Proportion refound species
Intercept	0.403**↓**	0.408	0.530**↓**	0.719↑	0.260**↓**
Temperature mean (°C)	*0.000*↑	*0.003***↓**	0.230↑	*0.001***↓**	*0.003*↑
Total precipitation (mm)	*0.004*↓	*0.018***↑**	0.124↑	0.564↑	0.630↑
Longest drought (days)	0.170↑	0.946	0.115**↓**	0.867**↓**	0.575**↓**
Freeze-thaw cycles	0.545**↓**	0.722	0.255↑	0.990↑	0.424↑
Depth (mm)	0.886↑	0.55	0.701**↓**	0.795**↓**	0.431**↓**
Slope (°)	0.198**↓**	0.37	0.976↑	*0.024*↑	0.527**↓**
Null deviance	67.8	48.1	34.79	44.49	40.4
Residual deviance	35.8	32	25.83	20.26	27.8
AIC (Akaike information criterion)	144.8	75	93.02	65.98	183.62

## Discussion

### Success of the originally intended vegetation

Several authors promote use of diverse species mixes to enhance the general performance and resilience of the vegetation by including species with complementary features (Isbell et al. 2011; Lundholm 2015). However, we found that a few species dominated cover and did not find many intended species at any site in either year. Similarly, in a previous study (Lönnqvist et al. submitted), we found that unintended species accounted for 69 ± 3% of the species present on green roofs in areas with a dry, cold subarctic climate, although the cover of spontaneous species was generally low. There was no significant further decline in vegetation cover between the survey years, indicating that early filtering of species occurred, likely through a combination of negative responses during the establishment phase in the local climate. This is consistent with our hypothesis that the standard green roof vegetation would not perform optimally in Nordic climatic conditions. For example, Emilsson 2008 saw a decline in total succulent cover; however, the trend was not as clear for the dominating mat forming species *S*. *acre* and *S*. *album.* Other studies outside Scandinavia have also detected a decline in species richness at sites with cold climates (Boivin et al. 2001). It should be noted that some species were planted on a few roofs; e.g. *H*. *cyaneum* and *S*. *oreganum* were only planted on four roofs in total. The roofs at the Oslo2 location were older than the other roofs (7–8 years), but showed no further decline (*p* < 0.05) in species richness compared to the younger (2–4 years old) roofs at the other sites. This suggests that most of the originally intended species failed to establish from the start, or rapidly declined during the first seasons (a conclusion corroborated by the lack of difference in this respect between the roofs in 2016 and 2017).

### Factors affecting survival and vegetation cover

#### Temperature and freeze-thaw cycles

We found that mean annual temperature strongly influenced the green roof vegetation dynamics (Table 6). Although many *Sedum* species are tolerant of cold conditions, their optimal temperature range for photosynthesis is 10–35 °C (Went 1953). At several of our study sites, there are limited numbers of days with temperatures within this optimal temperature range (yearly mean temperature Table 1). However, across our locations, there are variations in the length of the growing season and temperatures during both summer and winter, all with contrasting effects on succulent vegetation. Temperature was also positively correlated with accumulated precipitation across locations. Hence, vegetation responses across locations reflect composite effects of diverse factors, including negative effects on survival of episodes of winter precipitation, winter frost, and drought in spring or summer. Such filtering of species composition on green roofs by critical episodes has recognized importance (Bates et al. 2013; Vanstockem et al. 2019). We found that temperature was positively related to intended vegetation cover, but negatively related to unintended vegetation cover (Table 6). Since the amount of bare substrate was also negatively related to temperature, low temperatures could cause freezing injury to the intended vegetation, thereby leaving bare patches for unintended vegetation to colonize. In our study, the frequency of freeze-thaw cycles did not show any relationship with vegetation covers or survival of intended vegetation, possibly due to the vegetation being protected under snow cover during most of the freeze-thaw events. Mean annual temperature is most likely correlated to the number of days in the vegetation period at the sites; thus, mean temperature and possibly length of the vegetation period seem more important for the vegetation than the frequency of freeze-thaw cycles during the year (Table 6). In areas with cold climates, the water use of green roof vegetation should play a minor role for stormwater retention since evapotranspiration remains low (Johannessen et al. 2017). Johannessen et al. (2017) indicated that the mechanisms responsible for loss of vegetation cover are insufficiently understood, and highlighted the importance of critical episodes in order to better predict hydrological performance of the vegetation on cold climate green roofs. Accordingly, we found no impact of the number of freeze-thaw cycles, but did not account for effects of snow cover, due to lack of reliable data, which can certainly dampen effects of both freeze-thaw cycles and low temperatures (Boivin et al. 2001).

#### Precipitation and longest drought

To establish and maintain healthy green roof vegetation, appropriate supplies of available water are crucial (Young et al. 2017). Extensive green roofs with limited substrate depths are prone to water deficiency, which is the main reason why *Sedum* spp. (which have low water requirements) have long been preferred choices for green roof vegetation (Oberndorfer et al. 2007). We found that increases in accumulated annual precipitation had a significant negative effect on the intended (mostly succulent) cover, but not on the total cover of vascular plants or bare substrate (Table 6). Accordingly, parameters related to increasing plant available water in the substrate, e.g. reductions in roofs’ solar exposure, and increases in substrate depth or irrigation are known to facilitate the establishment of unintended vegetation (Dunnett et al. 2008). The intended vegetation consisted of drought-tolerant succulent species and the grass *F*. *ovina*, which are mostly stress tolerators (Grime et al. 2007). These plants thrive in stressful environments where resources such as water are limited. However, when resources become plentiful, they may be outcompeted by colonizing ruderal or competitive species that grow faster and have better resource allocation in such conditions (Dunnett 2015). Thus, at sites with many precipitation days and high annual rainfall, such as Bergen, other unintended colonizing species can establish and eventually compete with drought-tolerant intended vegetation (Fig. 3b). The positive relationship between total plant cover, including moss cover, and precipitation depth was consistent with expectations since many moss species thrive in moist conditions. According to Drake et al. 2018, mosses can inhibit germination of plants on green roofs, thereby potentially limiting the amount of spontaneous colonization. Here, relatively high spontaneous vegetation cover was found to coexist with moss in Bergen. We expected the longest dry period to be negatively related to survival and vegetation cover, but detected no such relationship (Table 6). However, it should be noted that during the two survey years, the lengths of the longest dry periods with no precipitation were relatively moderate and only exceeded more than 20 consecutive days at two locations (Table 3).

#### Roof design factors: water-holding layer, substrate, and slope

The depth of the substrate and its water-holding capacity have well-documented importance for vegetation cover and biomass (Durhman et al. 2007; Dunnett et al. 2008; Getter and Rowe 2008; Thompson et al. 2010; Thuring et al. 2010; Olly et al. 2011; Gabrych et al. 2016; Dusza et al. 2017). In stark contrast, we found that neither substrate depth nor water-holding layer thickness significantly affected the vegetation on our surveyed roofs (Table 6). However, all these roofs were of extensive type with shallow substrates (30–85 mm), and the importance of substrate depth decreases if the precipitation exceeds the actual evapotranspiration for most of the growing season. Thus, the small variations could obscure any effect of substrate depth on vegetation, at least in periods without extreme drought episodes, but in the drier locations in northern Sweden, there were some indications that the substrate was insufficiently deep to supply the vegetation with water. Previous studies of effects of the slope on green roof water retention have yielded inconsistent results, including indications that increasing the slope leads to greater water retention (VanWoert et al. 2005; Villarreal and Bengtsson 2005; Getter et al. 2007) and has no significant effect on retention (Bengtsson et al. 2005; Mentens et al. 2006; Liu et al. 2019). Theoretically it should negatively affect the water retention of green roofs (VanWoert et al. 2005; Getter et al. 2007), thereby reducing plant-available water and hence vegetation cover. Erosion by rain, wind, or snow is also more likely on sloped roofs, and the German green roof guidelines recommend additional watering of steeply sloped roofs to reduce plant mortality and subsequent erosion (FLL 2008). Few studies have investigated effects of slope on vegetation over time, but we found that roofs with greater pitch had significantly higher amounts of bare substrates (Table 6).

### Practical implications

Adaptable non-native species are often used in areas with harsh climates, but in this study, we also found that native *Sedum* cultivars performed relatively well (Table 5). Twenty percent of the roofs obtained a total vegetation cover (including all vascular plant and moss species) below the 80% cover that is recommended in the German guidelines for the construction of extensive green roofs (FLL 2008). Excluding the cover of unintended species and moss, the vegetation on most of the roofs (58%) did not reach this threshold, although they received standard maintenance, including fertilization. This shows that the target of 80% can be difficult to meet at sites with harsh climatic conditions, including short vegetation seasons and suboptimal temperatures. The cover of unintended vegetation was 10%, on average, but in some locations, it could dominate roofs and help efforts to reach the recommended cover targets. Replacement of *Sedum* by moss cover and unintended vegetation is an expected development in wet and cold areas, which could potentially contribute to services such as water retention and stability of the system, and warrants further investigation.

## Conclusion

We examined vegetation dynamics on standard *Sedum* mixes grown in vegetation mats on roofs in Scandinavian climates and effects of weather-related factors, and detected significant losses of species (relative to the original species lists) on all of 42 roofs, in nine locations, in the second survey year. We also detected substantial variation in vegetation cover on roofs at the same sites. In line with a first hypothesis, we found that the mean annual temperature of the preceding year was strongly positively related to the success of the intended vegetation. Conversely, the mean temperature was negatively related to the unintended vegetation cover, and the amount of bare substrate. In contrast to a hypothesis, mean annual precipitation was negatively related to the intended vegetation cover while it seemed to favour unintended vegetation. Opposed to our hypothesis, mean annual precipitation was negatively related to the intended vegetation cover. Design parameters had marginal impact on vegetation development, at least within the ranges of the parameters covered by the surveyed roofs, although roofs with high pitch have greater amounts of bare substrate. These results support our prediction that the standard *Sedum* vegetation would not perform optimally in all northern climatic conditions. Low mean annual temperature appears to be the main limiting factor for the success of the intended green roof vegetation. However, there was no detectable effect of low temperatures on moss cover, and the ability of unintended vegetation to thrive under such conditions reveals scope for improving vegetation selection for these conditions. The results also indicate that the species composition and substrate depth of green roofs should be carefully tailored to local conditions. Future research should explore the importance of species traits and the role of maintenance for green roof vegetation dynamics in cold areas.

## Supplementary information

ESM 1 (XLSX 59 kb)

## References

[CR1] Bates AJ, Sadler JP, Mackay R (2013). Vegetation development over four years on two green roofs in the UK. Urban For Urban Green.

[CR2] Bengtsson L, Grahn L, Olsson J (2005). Hydrological function of a thin extensive green roof in southern Sweden. Nord Hydrol.

[CR3] Boivin MA, Lamy MP, Gosselin A, Dansereau B (2001). Effect of artificial substrate depth on freezing injury of six herbaceous perennials grown in a green roof system. Horttechnology.

[CR4] Braskerud BC (2014) Green roofs and torrential rains, the effect of extensive roofs with sedum vegetation for reduced runoff after precipitation and snowmelt in Oslo, Norway (Norwegian). Oslo

[CR5] Butler C, Orians CM (2011). Sedum cools soil and can improve neighboring plant performance during water deficit on a green roof. Ecol Eng.

[CR6] Cook-Patton SC, Bauerle TL (2012). Potential benefits of plant diversity on vegetated roofs: a literature review. J Environ Manag.

[CR7] Drake P, Grimshaw-Surette H, Heim A, Lundholm J (2018). Mosses inhibit germination of vascular plants on an extensive green roof. Ecol Eng.

[CR8] Dunnett N (2015) Ruderal roofs. In: Green roof ecosystems. pp 233–255

[CR9] Dunnett N, Nagase A, Hallam A (2008). The dynamics of planted and colonising species on a green roof over six growing seasons 2001-2006: influence of substrate depth. Urban Ecosyst.

[CR10] Durhman AK, Rowe DB, Rugh CL (2007). Effect of substrate depth on initial growth, coverage, and survival of 25 succulent green roof plant taxa. HortScience.

[CR11] Dusza Y, Barot S, Kraepiel Y, Lata JC, Abbadie L, Raynaud X (2017). Multifunctionality is affected by interactions between green roof plant species, substrate depth, and substrate type. Ecol Evol.

[CR12] Dvorak B, Volder A (2010). Green roof vegetation for North American ecoregions: a literature review. Landsc Urban Plan.

[CR13] Emilsson T (2008). Vegetation development on extensive vegetated green roofs: influence of substrate composition, establishment method and species mix. Ecol Eng.

[CR14] Emilsson T, Rolf K (2005). Comparison of establishment methods for extensive green roofs in southern Sweden. Urban For Urban Green.

[CR15] Farrell C, Szota C, Williams NSG, Arndt SK (2013). High water users can be drought tolerant : using physiological traits for green roof plant selection. Plant Soil.

[CR16] FLL (2008) Guidelines for the planning, construction and maintenance of green roofing - green roofing guideline

[CR17] Gabrych M, Kotze DJ, Lehvävirta S (2016). Substrate depth and roof age strongly affect plant abundances on sedum-moss and meadow green roofs in Helsinki, Finland. Ecol Eng.

[CR18] Getter KL, Rowe DB (2006). The role of extensive green roofs in sustainable development. HortScience.

[CR19] Getter KL, Rowe DB (2008). Media depth influences Sedum green roof establishment. Urban Ecosyst.

[CR20] Getter KL, Rowe DB, Andresen JA (2007). Quantifying the effect of slope on extensive green roof stormwater retention. Ecol Eng.

[CR21] Getter KL, Bradley Rowe D, Cregg BM (2009). Solar radiation intensity influences extensive green roof plant communities. Urban For Urban Green.

[CR22] Grime JP, Hodgson JG, Hunt R (2007). Comparative plant ecology a functional approach to common British species.

[CR23] Isbell F, Calcagno V, Hector A, Connolly J, Harpole WS, Reich PB, Scherer-Lorenzen M, Schmid B, Tilman D, van Ruijven J, Weigelt A, Wilsey BJ, Zavaleta ES, Loreau M (2011). High plant diversity is needed to maintain ecosystem services. Nature.

[CR24] Jim CY (2017). Green roof evolution through exemplars: germinal prototypes to modern variants. Sustain Cities Soc.

[CR25] Johannessen BG, Hanslin HM, Muthanna TM (2017). Green roof performance potential in cold and wet regions. Ecol Eng.

[CR26] Köhler M, Poll PH (2010). Long-term performance of selected old Berlin greenroofs in comparison to younger extensive greenroofs in Berlin. Ecol Eng.

[CR27] Liu W, Feng Q, Chen W, Wei W, Deo RC (2019). The influence of structural factors on stormwater runoff retention of extensive green roofs: new evidence from scale-based models and real experiments. J Hydrol.

[CR28] Lönnqvist J, Viklander M, Blecken GT (submitted). Vegetation cover and plant diversity on cold climate green roofs. Manuscript re-submitted to Journal of Urban Ecology after minor revision

[CR29] Lundholm JT (2015). Green roof plant species diversity improves ecosystem multifunctionality. J Appl Ecol.

[CR30] Lundholm J, MacIvor JS, MacDougall Z, Ranalli M (2010) Plant species and functional group combinations affect green roof ecosystem functions. PLoS One 5. 10.1371/journal.pone.0009677 10.1371/journal.pone.0009677 PMC2837352 20300196

[CR31] MacIvor JS, Margolis L, Perotto M, Drake JAP (2016). Air temperature cooling by extensive green roofs in Toronto Canada. Ecol Eng.

[CR32] MacIvor JS, Sookhan N, Arnillas CA (2018). Manipulating plant phylogenetic diversity for green roof ecosystem service delivery. Evol Appl.

[CR33] Mentens J, Raes D, Hermy M (2006). Green roofs as a tool for solving the rainwater runoff problem in the urbanized 21st century?. Landsc Urban Plan.

[CR34] Monterusso MA, Bradley Rowe D, Rugh CL (2005). Establishment and persistence of Sedum spp. and native taxa for green roof applications. HortScience.

[CR35] Oberndorfer E, Lundholm J, Bass B, Coffman RR, Doshi H, Dunnett N, Gaffin S, Köhler M, Liu KKY, Rowe B (2007). Green roofs as urban ecosystems: ecological structures, functions, and services. Bioscience.

[CR36] Olly LM, Bates AJ, Sadler JP, MacKay R (2011). An initial experimental assessment of the influence of substrate depth on floral assemblage for extensive green roofs. Urban For Urban Green.

[CR37] Sailor DJ (2008). A green roof model for building energy simulation programs. Energy Build.

[CR38] Speak AF, Rothwell JJ, Lindley SJ, Smith CL (2013). Reduction of the urban cooling effects of an intensive green roof due to vegetation damage. Urban Clim.

[CR39] Stovin V, Poë S, De-Ville S, Berretta C (2015). The influence of substrate and vegetation configuration on green roof hydrological performance. Ecol Eng.

[CR40] Thompson K, Petchey OL, Askew AP, Dunnett NP, Beckerman AP, Willis AJ (2010). Little evidence for limiting similarity in a long-term study of a roadside plant community. J Ecol.

[CR41] Thuring CE, Berghage RD, Beattie DJ (2010). Green roof plant responses to different substrate types and depths under various drought conditions. Horttechnology.

[CR42] Tran S, Lundholm JT, Staniec M, Robinson CE, Smart CC, Voogt JA, O'Carroll DM (2019). Plant survival and growth on extensive green roofs: a distributed experiment in three climate regions. Ecol Eng.

[CR43] Vanstockem J, Somers B, Hermy M (2019). Weeds and gaps on extensive green roofs: ecological insights and recommendations for design and maintenance. Urban For Urban Green.

[CR44] VanWoert ND, Rowe DB, Andresen JA (2005). Green roof stormwater retention: effects of roof surface, slope, and media depth. J Environ Eng.

[CR45] Vasl A, Shalom H, Kadas GJ, Blaustein L (2017). Sedum—annual plant interactions on green roofs: facilitation, competition and exclusion. Ecol Eng.

[CR46] Villarreal EL, Bengtsson L (2005). Response of a Sedum green-roof to individual rain events. Ecol Eng.

[CR47] Warton DI, Hui FKC (2011). The arcsine is asinine: the analysis of proportions in ecology. Ecology.

[CR48] Went FW (1953). The effect of temperature on plant growth. Annu Rev Plant Physiol.

[CR49] Williams NSG, Lundholm J, Scott Macivor J (2014). Do green roofs help urban biodiversity conservation?. J Appl Ecol.

[CR50] Young TM, Cameron DD, Phoenix GK (2017). Increasing green roof plant drought tolerance through substrate modification and the use of water retention gels water retention gels. Urban Water J.

